# Vacteens.org: A Mobile Web app to Improve HPV Vaccine Uptake

**DOI:** 10.3389/fdgth.2021.693688

**Published:** 2021-08-25

**Authors:** W. Gill Woodall, Gregory Zimet, Alberta Kong, David Buller, Jeannyfer Reither, Lance Chilton, Valerie Myers, Randall Starling

**Affiliations:** ^1^Klein Buendel Inc., Golden, CO, United States; ^2^Division of Adolescent Medicine, Department of Pediatrics, Indiana University-Purdue University Indianapolis (IUPUI) Center for HPV Research, Indiana University, Indianapolis, IN, United States; ^3^Department of Pediatrics, The University of New Mexico Health Sciences Center, Albuquerque, NM, United States; ^4^Center on Alcohol, Substance Use and Addictions (CASAA), The University of New Mexico, Albuquerque, NM, United States

**Keywords:** HPV, vaccination uptake, digital intervention, adolescents, parents

## Abstract

U.S. HPV vaccine uptake remains below the Healthy People 2030 goal of 80% series completion. Parental concerns and misinformation about the efficacy and safety of the Human Papillomavirus (HPV) vaccine remain, and may be addressed by digital interventions tailored to their concerns. Reported here are results from a small scale randomized trial testing a mobile web app for parents and their adolescent daughters (ages 11–14 years) encouraging HPV vaccination in New Mexico, an ethnically-diverse U.S. state.

**Methods:** A clinic-cluster randomized trial where pediatric clinics (*n* = 9) were recruited and randomized, and parent-adolescent pairs (*n* = 82) within clinics received either the *Vacteens.org/Vacunadolescente.org* mobile web app or Usual and Customary (UC) HPV Vaccination information. Parents completed online surveys at baseline and 3-months. Daughters' HPV vaccine data were collected from the New Mexico State Immunization Information System 1 year post baseline.

**Results:** Three month survey results found *Vacteens.org/Vacunadolescente.org* parents to have higher positive HPV vaccine beliefs, informed decision making, intent to vaccinate and vaccine confidence outcomes than UC parents. HPV vaccine data found higher first dose HPV vaccination (Pearson χ^2^ = 6.13, *p* = 0.013, *Vacteens.org/Vacunadolescente.org* group 59.4%, UC group 40.6%), and higher HPV vaccination series completion (Pearson χ^2^ = 6.49, *p* = 0.011, *Vacteens.org/Vacunadolescente.org* group 68.4%, UC group 31.6%).

**Conclusions:** The small trial results showed the *Vacteens.org/Vacunadolescente.org* web app prompted positive vaccine-related attitudes and beliefs, and more HPV vaccination initiation and series completion. Mobile web apps can make decision-making tools for HPV vaccination widely available on digital platforms, reducing vaccine hesitancy, and confusion and increase HPV vaccine uptake.

## Introduction

In the U.S. uptake of Human Papillomavirus (HPV) vaccine remains far below the Healthy People 2030 goal of 80% series completion (1). Nationally, 54.2% of adolescents aged 13–17 were up-to-date for the Human Papillomavirus (HPV) vaccine in 2019 [females 56.8%; males: 51.8% (2)]. In New Mexico, HPV vaccination completion for this adolescent age range also remains low (59.8%).

While a number of factors may account for this less than desirable vaccine uptake, parental concerns, and misinformation about the efficacy and safety of HPV vaccine remain barriers to reaching public health vaccination goals (1, 2). Vaccine initiation is affected by health beliefs (e.g., vaccine knowledge; importance of preventive vaccinations; side effects concerns), while vaccine completion [2 doses if started by age 14; 3 doses if after age 15 (3)] is affected both by logistical barriers (e.g., forgetting; scheduling difficulties; child care; travel time; physician hesitancy) *and* health beliefs (4–10). Health beliefs are amenable to health education interventions. Research indicates there is a great deal of (1) confusion and uncertainty about HPV vaccine and (2) concomitant misinformation about HPV vaccine, who it is meant for, and the conditions under which it is maximally effective.

Identifying effective strategies to improve HPV vaccination rates is a priority for the Centers for Disease Control and Prevention (CDC) (11) and the World Health Organization (WHO) (12). Physician and clinic-based interventions have shown some positive effect on vaccine uptake (13–15), however parental concerns and hesitancy remain barriers to HPV vaccine acceptance. Given that clinicians have limited time to interact with parents during primary care pediatric and adolescent visits, parental barriers to HPV vaccination may ideally be addressed by digital interventions (in this case, smartphone applications) that are tailored to their concerns, especially since virtually all U.S. adults under age 50 use the Internet (16). As of 2019, there were few differences in Internet use by gender, ethnicity, or urban/rural status, with use exceeding 85% in all groups. Nearly 1 in 5 adults under age 50 of both genders use their smartphone for online access, with Hispanics and rural adults showing the highest use of this cellular Internet access (16).

Reported here are findings from a randomized trial on a smartphone web app for parents and adolescent girls (ages 11–14) that was intended to encourage HPV vaccination in New Mexico, an ethnically-diverse U.S. state. The trial tested the following hypotheses:

H1: Parents assigned to the *Vacteen/Vacunadolescente* mobile web app will express more favorable HPV vaccine beliefs, informed decision making, intent to vaccinate for HPV, self-efficacy for HPV vaccination, and benefits and risks of HPV vaccination for their daughters than parents assigned to the Usual and Customary (UC) Information control group.

H2: More daughters of parents assigned to the *Vacteen/Vacunadolescente* mobile web app will initiate and complete the HPV vaccination series than daughters of parents assigned to the UC Information control group.

## Materials and Methods

### *Vacteens/Vacunadolescente* Mobile Web app

The current project translated an earlier version of the website (the *GoHealthyGirls* website) to a mobile app platform, *Vacteens/Vacunadolescente.org*, and provided both English and Spanish versions. Mobile web apps are mobile device sensitive web sites designed to function like native operating system (Android or iOS) apps, but avoid the problems of operating system exclusivity. They maintain app functionality across mobile platforms, and are more easily updatable should that be required. The web app developed in this project was an informed decision-making website for parents and adolescent daughters (ages 11–14) that employed both Informed Decision Making [IDM, (17)] and Diffusion of Innovations Theory [DOI, (18)] principles in messaging. Informed Decision Making theoretical principles indicate focusing on beliefs and attitudes parents hold that constitute barriers to vaccination, often based on misinformation, is important for vaccination messaging. At the same time, DOI theory suggests that treating vaccination as an innovation is useful, and messaging on the simple, compatible with beliefs, and trialable characteristics of HPV vaccination will improve vaccine adoption. The website was programmed as a web app for mobile devices with open non-linear navigation. It had a video introduction by a well-known New Mexican pediatrics physician, a Vaccine FAQ section, and five modules: (1) *Get Answers!* about HPV and vaccines, risks and side effects of the vaccine, risks of HPV, benefits of HPV vaccination and organizations recommending HPV vaccination; This module addressed the concerns and misinformation parents have about the HPV vaccine. (2) *Let's Talk* on the communication process around vaccination, including a video simulation on how to talk with your daughter about HPV vaccination, guidelines for talking to family members and physician about HPV vaccination; This module provides communication examples to be modeled by parents discussing vaccination with their daughters, and suggestions for discussions with other family members and the health care provider. (3) *Vaccine How-To* with instructions for making an HPV vaccination appointment; This module contains location and appointment tools for getting vaccinated that parents and teens can use for vaccination action plans. (4) *Teen Tools* with interactive games for teens, i.e., HPV Challenge Quiz, and HPV Myth vs. Truth swiping game; This module contains interactive and engaging activities for both teens and parents, all focused on providing accurate and motivating information about the HPV vaccine. (5) *We're Ready* providing email and texting HPV vaccination reminder systems to promote completion of the vaccination series, accessible from any page in the web app. This module provides notification and reminder tools for the second and possible third dose of the HPV vaccine. While the predominant messaging in the web app was focused on parents, their daughters also had specific content for their use (*Teen Tools*). Overall, the web app content and language were designed to encourage both parent and adolescent use, both separately and together.

#### Development of the Vacteens/Vacunadolescente Web app

The *Vacteens/Vacunadolescente* web app was systematically developed through developmental research. The *GoHealthyGirls* project (funded by the National Institute of Allergy and Infectious Diseases—U19 AI084081) employed DOI and related IDM research to guide the iterative development of a website for parents of young female adolescent daughters (ages 11–14). It was systematically developed via parent and adolescent focus groups, navigability and usability tests (19), and a beta test with an ethnically-diverse sample of parents and daughters in New Mexico (20). Results indicated the website to be easy and enjoyable to use and had clear impact on theoretical antecedents to HPV vaccine uptake (e.g., attitudes, risk perceptions, consequences, self-efficacy, and intent to get daughter vaccinated).

### Clinic-Cluster Randomized Trial

A clinic-cluster randomized trial was conducted in New Mexico. Pediatric clinics (*N* = 9) were recruited and randomized to receive either the *Vacteens/Vacunadolescente.org* web app (*n* = 5) or the Usual and Customary (UC) HPV vaccination information (*n* = 4) available from the Centers for Disease Control and Prevention (CDC) online. Clinics were randomized before recruitment of parents and daughters, but physicians and clinic staff were kept blind to treatment assignment. Parents were recruited from clinics by project staff via telephone contact. Inclusion criteria for the trial were to be parents of an 11–14 year old daughter who had not yet received HPV vaccination. Exclusion from the trial was to have had the parent's daughter already vaccinated for HPV. Participants were qualified and registered for the project on a project registration website and provided online informed consent, daughter assent, and HIPAA waiver to access daughter vaccination records from the New Mexico State Immunization Information System (NM-SIIS). All project procedures were reviewed and approved by the University of New Mexico Main Campus Institutional Review Board. Parents of daughters aged 11–14 and the daughters themselves were recruited from participating pediatric clinics (*N* = 82 parent-daughter pairs). Parents were assessed via online survey at baseline and 3-month post-baseline assessments. Once parents were qualified, consented and registered for the project, and had completed the baseline assessment (see Parent Surveys on Antecedents to Vaccination), based on their clinic randomization, they were provided a link to either the *Vacteens/Vacunadolescente.org* web app or the UC CDC web link. These links remained active for the year-long project for parents and their adolescents to browse. HPV Vaccine uptake data available from NM-SIIS for daughters of participating parents were collected at 1-year post-baseline.

### Parent Surveys on Antecedents to Vaccination

Parents were assessed by online surveys via QuestionPro survey software at baseline and 3-month assessment points. The surveys measured participants' demographic characteristics (gender, age, race/ethnicity, language preference, educational level, and sociodemographic status), and HPV related variables, including: HPV knowledge [Cronbach's α = 0.60 (21)], HPV vaccine attitudes, e.g., “It is important to get vaccines because they prevent disease,” [α = 0.89 (22, 23)], perceived daughters' risk of HPV, e.g., “Infection with HPV can lead to serious illness,” [α = 0.73 (19, 23)], beliefs about HPV and HPV vaccination, e.g., “The HPV vaccine is effective at preventing cervical cancer,” [α = 0.91 (24)], intention to have daughter vaccinated (single item), “If you were asked to make a decision right now about getting your daughter her first HPV shot, what would you decide?,” HPV informed decision making, e.g., “I know which options are available to me regarding the HPV vaccine [α = 0.98 (25)].

### Vaccination Records

Vaccination records were acquired by matching parent identification information to the NM-SIIS database. Record acquisition was performed by an honest broker, who was blind to clinic and parent randomization status. Participants' parents last name, adolescents last name, first name and birthdate were used as matching variables in vaccination record acquisition. First and second shot completion data were recorded from participant daughters' records.

### Data Analysis

Statistical analyses were conducted with SPSS ver. 27. Both descriptive statistics and inferential tests for group differences were calculated. To evaluate differences in vaccine beliefs and attitudes, one-tailed *t*-tests were used. We chose to use one-tailed tests because our hypotheses were directional and a less conservative approach to analysis was believed to be appropriate for this relatively small sample evaluation [cf. Kirk (26)]. The effect of intervention group on HPV vaccine uptake was determined via non-parametric Chi-Square analyses, as recommended by Williams and Monge (27). Analyses were conducted on an unadjusted for clinic cluster effects basis after determining the Intraclass Correlation (ICC) within clinics for participant baseline HPV knowledge variables was near zero.

## Results

### Participants

Parent participants (*N* = 82) were 92.5% female, 38.5% Hispanic, 6.2% American Indian/Native Alaskan, 1.2% Asian, and 37.8% Caucasian, with 12.3% unspecified and 3.7% missing information. The average age of parent participants was 38.96 years (SD = 9.64), and average age of daughter participants was 12.05 years (SD = 1.08). Educational attainment was 3.8% 11th grade or less, 33.8% high school diploma or G.E.D., 25.0% Associates degree, 18.8% Bachelor's degree, 8.8% Masters degree, 1.3% Doctorate degree, and 7.5% Other Professional degrees. Language preference for parent participants was 96.3% English, with 16.3% additionally speaking Spanish, and 6.3% additionally speaking a Tribal Language.

### Hypothesis 1: Vaccine Antecedents

Three-month follow-up surveys were completed by 38% (*n* = 31) of the study sample. The remainder of the participants were not available to be surveyed due to early termination of the trial by the funding agency. A review of all baseline participants found no statistically significant differences (Pearsons χ^2^-tests) in demographics between participants who did and did not respond to 3-month assessments. Analyses of the available 3-month assessment data for parents found several statistically-significant differences between the *Vacteens.org/Vacunadolescente* and UC Information participants. Planned *t*-tests (*p* < 0.05, one-tailed, *df* = 31) revealed significant between group differences in the predicted direction for HPV vaccine beliefs [*t*_(31)_ = 3.87, *p* = 0.001]; Informed Decision Making [*t*_(31)_ = 4.29, *p* = 0.047]; parents in the *Vacteens.org/Vacunadolescente* were also more likely to intend to vaccinate their daughters right away than later or not at all (Pearson χ^2^ = 5.70, *p* = 0.05. Cohen's *d* = 0.94, *OR* = 6.23). In addition, parents in the *Vacteens.org/Vacunadolescente* group were significantly more confident about their vaccination choices (Informed Decision Making; Pearson χ^2^ = 4.28, *p* = 0.03, *d* = 0.80, *OR* = 4.92), and a trend toward being more aware of the benefits and risks of vaccination (Pearson χ^2^ = 2.97, *p* = 0.08).

### Hypothesis 2: Vaccination Outcomes

HPV vaccine uptake data from the NM-SIIS database was obtained for all daughters of parents enrolled in the trial (*n* = 82). A review of first shot date and date of entry into the study determined that some daughters (*n* = 13) had received their initial HPV vaccinations prior to study, and thus were not qualified to participate. Data for these cases were excluded from the analysis; exclusion occurred equally from the *Vacteens.org/Vacunadolescente* (*n* = 7) and UC Information (*n* = 6) groups, leaving a final *N* = 69 for analysis. Analyses of first dose data revealed a significant treatment group difference (Pearson χ^2^ = 6.13, *p* = 0.013, *d* = 0.62, *OR* = 3.45), such that rate of HPV vaccination initiation in the *Vacteens.org/Vacunadolescente* condition (59.4%) was 18.8% higher than the UC Information condition (40.6%). Further, HPV vaccination series completion in the *Vacteens.org/Vacunadolescente* group was statistically-significantly higher (Pearson χ^2^ = 6.49, *p* = 0.011, *d* = 0.64, *OR* = 4.53) (68.4%) compared to the UC group (31.6%), an absolute increase of 36.8%.

## Discussion

The results of this trial indicated that the *Vacteens.org/Vacunadolescente* mobile web app bolstered parents' positive HPV vaccine beliefs, Informed Decision Making, and intentions to vaccinate, and most importantly led to higher levels of vaccine initiation (i.e., first dose) and series completion (i.e., second dose). The small sample of parent-daughter pairs may limit confidence in the outcome, but the effect sizes and odds ratios are in the moderate range, suggesting a substantial effect of the *Vacteens.org/Vacunadolescente* web app that would potentially make large in-roads into vaccine uptake when distributed widely.

There are a number of implications that the study results suggest for deploying the *Vacteens.org/ Vacunadolescente* mobile web app. First, it could be used in conjunction with a pediatric clinic practice, where physicians recommend use of the mobile web app prior to well-child visits, sports physicals, or vaccination appointments. Parents who browse the app may make informed decisions about vaccination before the visit and be ready for vaccine initiation, saving valuable time in the doctor-patient interaction, time that is already at a premium. It also may make it more comfortable for providers to talk with parents about HPV vaccination, knowing that the topic was already presented and many of parents' concerns were covered in the mobile web app. Further, tools provided in the *Vacteens.org/Vacunadolescente* mobile web app, like the text and email follow-up reminders, could make vaccine dose completion more likely, as our data show. Thus, in combination with presumptive recommendations (13) by pediatricians for HPV vaccination and other clinic-based techniques, the use of *Vacteens.org/Vacunadolescente* might substantially improve vaccine uptake in this age range during clinical encounters.

A second possibility is that the *Vacteens.org/Vacunadolescente* mobile web app could be used by parents independent of medical clinics. Many vaccinations of all kinds now occur outside of pediatric or other medical practices in, for example, pharmacies oriented to vaccine provision. This lessens the reliance on pediatricians and other medical providers for advice and recommendation for the HPV vaccination, and for vaccine provision. Other entities involved in vaccination, such as state health departments, school health officials, and pharmacy chains, could promote the use of the *Vacteens.org/Vacunadolescente* mobile web app to increase HPV vaccination initiation and completion at whatever provider to which parents have access in communities. Further, parents of adolescents in this age range are often excessively busy, leading to a drop in the frequency of having their child seen by a pediatrician or medical professional, often limited to as little as once a year for a well-child checkup prior to the start of the school year. Again, these factors may make the use of *Vacteens.org/Vacunadolescente* mobile web app outside of the clinic viable as a way to support and promote vaccination independent of clinical practice.

The present investigation carries some limitations. The small sample size is a limitation, and further research will be needed to confirm the impact of the web app on vaccine uptake and related variables. The findings are also limited to young adolescent girls ages 11–14, even though HPV vaccination is recommended for boys. We are currently conducting a trial with a version of the web app tailored to parents of young male adolescents in the same 11–14 years of age. The loss of some parents due to already having had their daughter vaccinated, is of some concern; however, the results remained statistically significant with moderate effect size. It seems that some parents were simply not sure as to whether they had their daughter vaccinated for HPV. Paper-based methods for tracking vaccination, especially in adolescence, are now rarely used, and parents may lose track of vaccination instances. Currently, the New Mexico Department of Health provides an online portal where parents can search for their child's vaccination record (a number of states have begun to adopt this technology), but parents may be unaware of this resource. The loss of a substantial amount of 3-month follow-up survey data due to trial termination by the funding agency is a limitation to the study. The loss of these data certainly limits conclusions available from the study survey analyses. In retrospect, the trial termination (due to insufficient progress in clinic recruitment) is regrettable given the promising data that the investigation was able to obtain. The location of the trial in New Mexico may limit its generalizability due to its ethnic mix, containing predominately Hispanic and Native American minority participants. Whether the mobile web app would be just as effective with African American parents or other minority group parents is unknown. A final limitation is the young age of the sample (11–14 years). HPV vaccination is recommended for individuals up to age 26 and we cannot be certain that the *Vacteens.org/Vacunadolescente* mobile web app would convince parents of older daughters (those ages 16+) to seek the HPV vaccine for them. The *Vacteens.org/Vacunadolescente* web app focused its messaging on this younger 11–14 years age range, and parents of older teens (females and males) may need somewhat different messaging that is sensitive to older teens having more agency in vaccination decisions.

The results of this investigation suggest that mobile web app technology, systematically developed for ease and convenience of use on mobile and other computing devices and guided by DOI and IDM theories of health behavior, that communicates about parents' concerns, lack of information, and misinformation parents hold regarding the HPV vaccination, can substantially improve HPV vaccine uptake. A recent review of social media and mobile technology interventions to improve HPV vaccine uptake (28) indicates that text message, e-mail, phone contact and social media groups can improve HPV vaccine uptake. This investigation adds mobile web applications to the list of digital techniques for vaccine uptake improvement. To prevent a variety of HPV-related cancers, the use of digital communication outside clinics for promoting HPV vaccination is well worth considering, especially as many parents' lives are replete with digital messaging and mobile devices.

## Data Availability Statement

The raw de-identified data supporting the conclusions of this article will be made available by the authors, without undue reservation.

## Ethics Statement

The studies involving human participants were reviewed and approved by University of New Mexico Main Campus Institutional Review Board. Written informed consent to participate in this study was provided by adult participants, and adolescent assent was provided by adolescent participants' legal guardian/next of kin.

## Author Contributions

WW oversaw research project in all aspects, conducted data analysis, and major amount of manuscript writing. GZ was involved in message construction, measurement, and manuscript writing. AK was involved in message construction, clinic recruitment, and manuscript writing. DB was involved in message construction, web app development, testing, and manuscript writing. JR was message language translation and web app development. LC was involved in clinic recruitment, message construction, and vaccine data. VM was involved in message development and measure assessment. RS was involved in clinic recruitment. All authors contributed to the article and approved the submitted version.

## Conflict of Interest

WW, DB, JR, and VM were employed by company Klein Buendel Inc. Outside of the present work GZ has served as an external advisory board member for Merck and Moderna and as a consultant to Merck. In addition, he has received investigator-initiated research funding from Merck administered through Indiana University. The remaining authors declare that the research was conducted in the absence of any commercial or financial relationships that could be construed as a potential conflict of interest.

## Publisher's Note

All claims expressed in this article are solely those of the authors and do not necessarily represent those of their affiliated organizations, or those of the publisher, the editors and the reviewers. Any product that may be evaluated in this article, or claim that may be made by its manufacturer, is not guaranteed or endorsed by the publisher.
